# Morphometry of Fetal Liver From Human Fetuses Between 12-36 Weeks Gestational Age

**DOI:** 10.7759/cureus.24060

**Published:** 2022-04-12

**Authors:** Kavita Modi, Amarjyoti Chaturvedi, Akhalaq Ahmad, Pooja Bhadoria

**Affiliations:** 1 Anatomy, All India Institute of Medical Sciences, Rishikesh, Rishikesh, IND

**Keywords:** anatomy, morphology, morphometry, gestation age, liver, fetus

## Abstract

Background of the study

To assess the prenatal development of the human liver between 12-36 weeks of gestational age by measuring morphometric parameters using conventional autopsy and to evaluate the morphometric parameters of the human fetus and its liver and their correlation to predict the gestation age.

Materials & methods

The present study was conducted in the Department of Anatomy, All India Institute of Medical Sciences, Rishikesh, India, on 33 normal fetuses of gestational age 12-36 weeks collected from the Department of Obstetrics and Gynecology of the same institute. which were classified into five groups: A (12-16 weeks), B (17-21 weeks), C (22-26 weeks), D (27-31 weeks), and E (32-36 weeks). The parameters measured were liver weight, liver volume, transverse diameter, sagittal diameter, vertical length, length, and width of all four lobes of the liver, i.e., right, left, caudate and quadrate lobe. Also, general morphometric parameters of the fetuses were measured like fetal body weight, crown-rump length, crown-heel length, biparietal diameter, head circumference, chest circumference, abdominal circumference, hand length, foot length, inner inter-canthal distance, outer inter-canthal distance. The obtained data were statistically analyzed using ANOVA, and Pearson’s correlation was assessed.

Results

There was a statistically significant increase amongst all the fetal general parameters and parameters of liver except bi-parietal diameter, p-value <0.001. The bi-parietal diameter was weakly statistically significant correlated with all other parameters except with chest circumference, crown-heel length, length and width of caudate lobe, and the width of the quadrate lobe and left lobe where it was statistically non-significant.

Conclusion

Bi-parietal diameter is a statistically non-significant parameter to calculate gestation age. The knowledge of morphological features and normal limits of dimensions of the liver with respect to gestational age is a reliable reference to help to prevent misdiagnosis of various pathological conditions of the liver like cirrhosis, hepatomegaly, fetal anemia, intrauterine growth retardation, congenital anomalies like Down's Syndrome, etc.

## Introduction

In adults, the liver is the largest internal visceral organ present in right hypochondrium, epigastrium, and left hypochondrium [1]. It appears in the third week of the embryonic stage and grows rapidly from the fifth to tenth week of gestation, occupying a substantial space of the abdominal cavity throughout the intrauterine life. The liver represents 10% of total body weight at 10 weeks. Initially, both right and left lobes have same size but then, the right lobe preferably becomes large [2]. In postnatal life, it contributes to 4-5% of infant body weight, and then reduces to nearly 2% in puberty, approximately 1500 g in weight [3]. Maternal diabetes affects the growth of fetal liver in the way that liver volume increases by 20% at every gestation week than normal controls [4]. In a study by Murao et al., the authors concluded that the measurements of fetal liver size aids in assessing the intrauterine growth of fetuses [5]. Most of the research on morphology and morphometry of human fetal liver in normal and abnormal pregnancies is based on radiology interventions. In this modern era of widespread acceptance for MRI and ultrasonography, the use of conventional autopsy for quantitative evaluation of fetal organs is a gold standard method. Previous studies indicate a straight relationship between parameters of fetal liver and its general morphometric measurements of fetal body and derive a conclusion with its gestational age [6]. The current study aims to look at the comprehensive gross morphological aspects of the human fetal liver using morphometric analysis and its link between gestational ages. The evaluation of fetal morphometric parameters and liver dimensions is crucial for estimating fetal gestational age, detecting anatomical variations, and identifying congenital defects in branches such as anatomy, surgery, forensic sciences, medicine, radiology, pediatrics, obstetrics, and fetopathology.

## Materials and methods

The current study was an institutional-based, observational, cross-sectional study conducted by the Department of Anatomy in collaboration with the Department of Obstetrics & Gynecology, All India Institute of Medical Sciences, Rishikesh, Uttarakhand, India. Assuming the expected population SD to be 5.5, and employing t-distribution to estimate sample size, the study required sample size of 33, to estimate a mean with 95%CI and absolute precision of 2 [7].

The present study was done on 33 normal fetuses (male-22, female-11) that were collected immediately after delivery and received with written consent from the mother and legal guardian. Ethical clearance was taken from the Institutional Ethical Committee (Reg No.: ECR/736/Inst/UK/2015/RR-18) of All India Institute of Medical Sciences, Rishikesh, Uttarakhand, India. The rules and standard guidelines for the disposal of human anatomical wastes were strictly followed [8].

Inclusion and exclusion criteria

Fetuses from induced abortions (under MTP Act 1971), fetuses from healthy mothers, and fetuses without any gross congenital abnormality were included in the study. Decomposed fetuses, fetuses with any gross congenital abnormality, or fetuses from mothers with any illness during pregnancy were excluded.

Fetal autopsy

**Figure 1:**
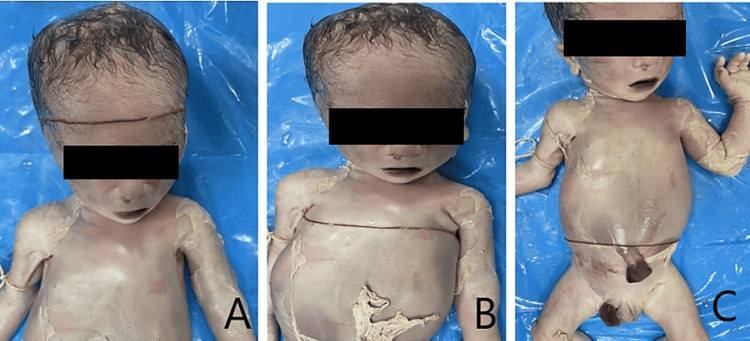
Panel A, Panel B, and Panel C show the measurement of head circumference, chest circumference, and abdominal circumference, respectively.

**Figure 2:**
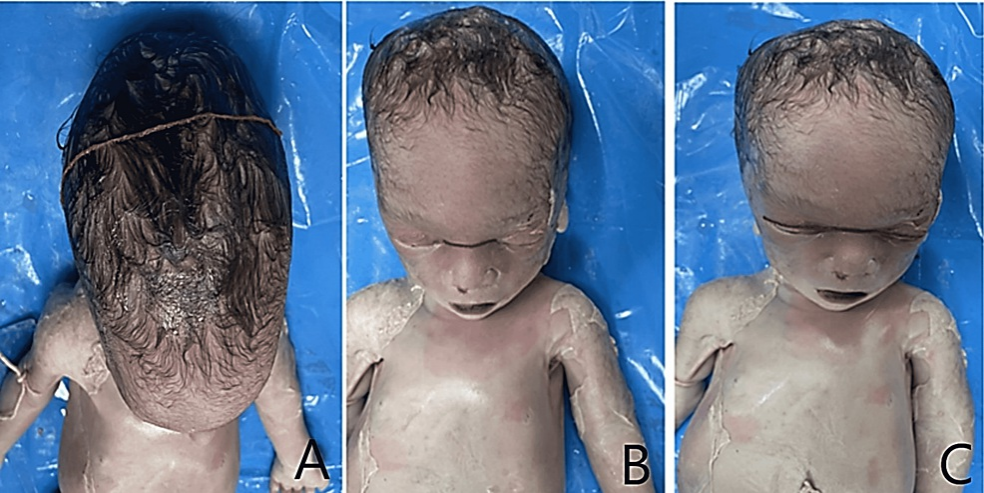
Panel A, Panel B, and Panel C show the measurement of biparietal diameter, inner intercanthal distance, and outer intercanthal distance, respectively.

**Figure 3:**
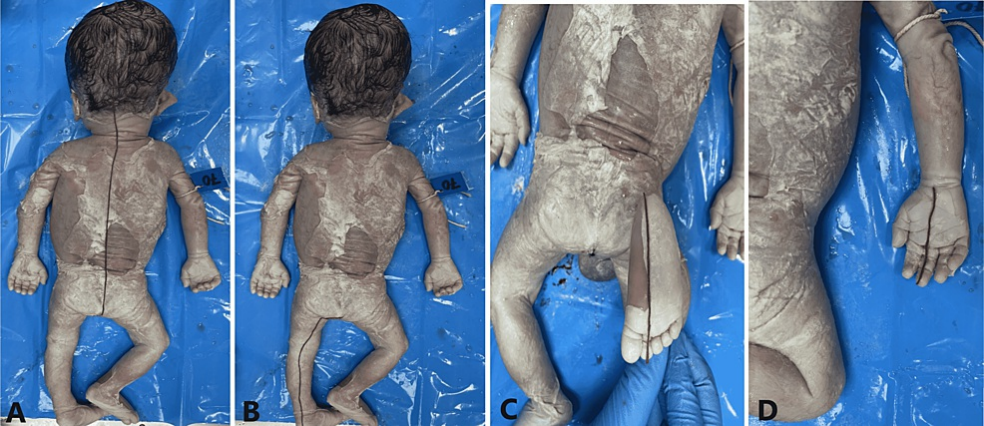
Panel A, Panel B, Panel C, and Panel D show the measurement of crown-rump length, crown-heel length, foot length, and hand length, respectively.

**Figure 4:**
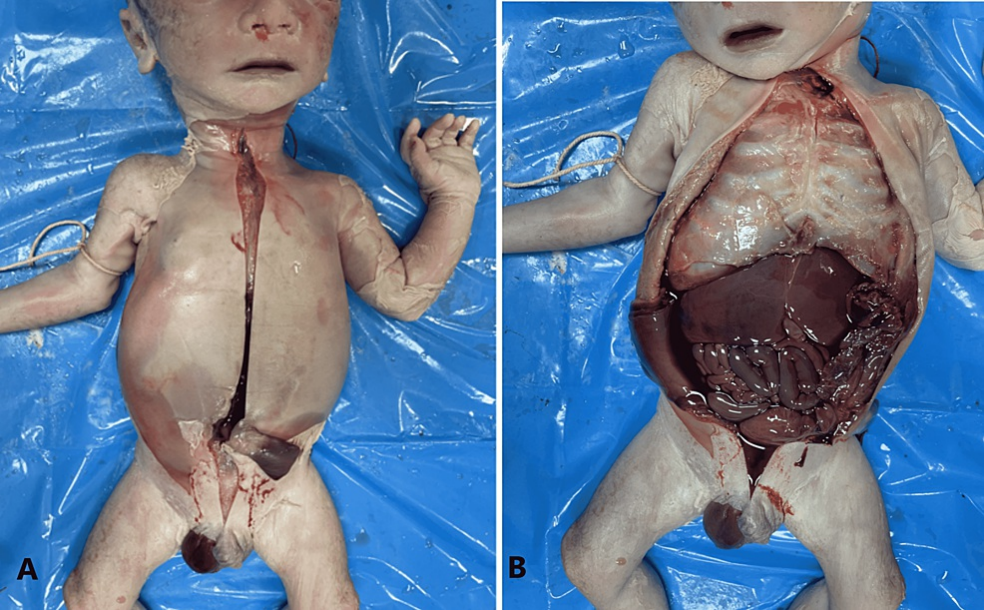
Panel A shows a midline incision extending from suprasternal notch to pubic symphysis. Panel B shows the exposed abdominal viscera.

**Figure 5:**
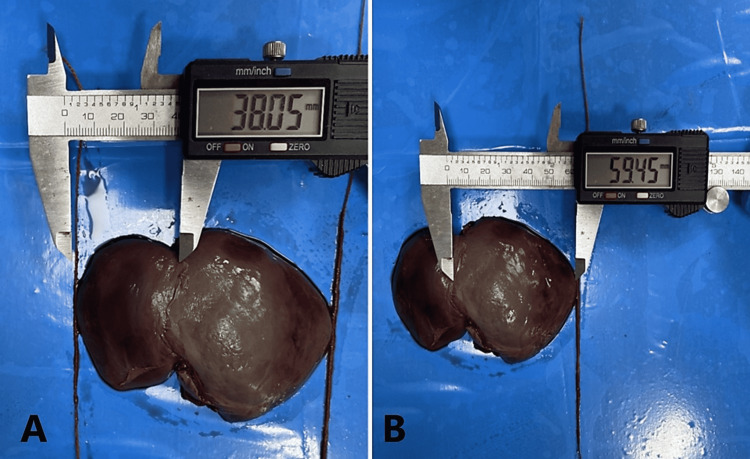
Panel A shows the measurements of width of left lobe of human fetal liver in anterior projection. Panel B shows measurement of width of right lobe of human fetal liver in anterior projection.

**Figure 6:**
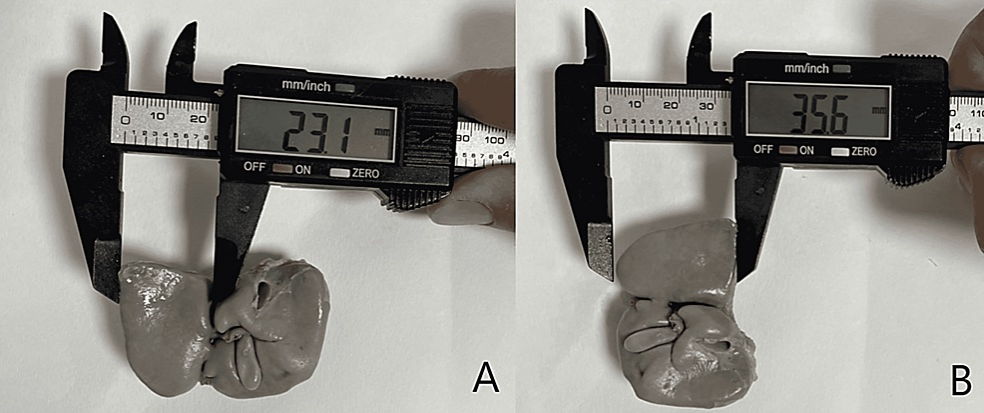
Panel A and Panel B show the measurement of width and length of left lobe on posterior surface of human fetal liver. In Panel B, liver has been rotated 90° right.

**Figure 7:**
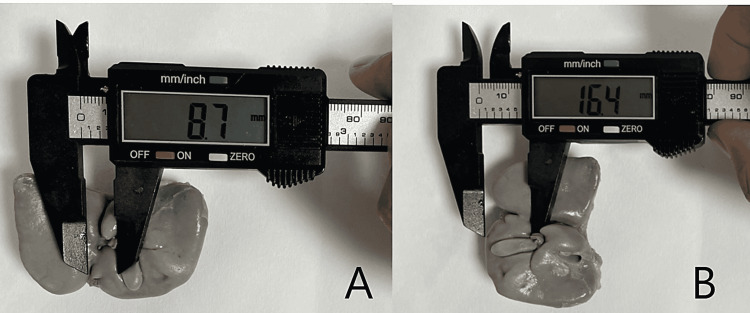
Panel A and Panel B show the measurement of width and length of quadrate lobe on posterior surface of human fetal liver. In Panel B, liver has been rotated 90° right.

**Figure 8:**
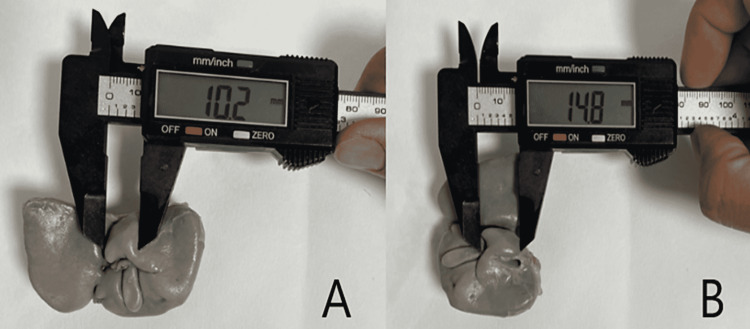
Panel A and Panel B show the measurement of width and length of caudate lobe on posterior surface of human fetal liver. In Panel B, liver has been rotated 90° right.

**Figure 9:**
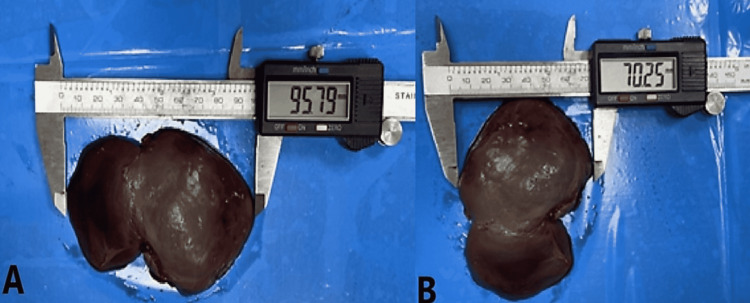
Panel A and Panel B show the measurement of transverse diameter and vertical length on anterior projection of human fetal liver respectively. In Panel B, liver has been rotated 90°left.

The gestational age of the obtained fetuses ranged from 12-36 weeks, confirmed by ultrasound. They were classified into five groups: group A (12-16 weeks), group B (17-21 weeks), group C (22-26 weeks), group D (27-31 weeks), and group E (32-36 weeks). The fetuses were preserved in 10% formalin. General morphometric parameters such as fetal body weight (FBW), head circumference (HC), chest circumference (CC), abdominal circumference (AC), bi-parietal diameter (BPD), inner inter-canthal distance (ICD) and outer inter-canthal distance (OCD), hand length (HL), foot length (FL), crown-rump length (CRL), and crown-heel length (CHL) were measured by using cotton thread and ruler (Figure 1, Figure 2, Figure 3). They were further autopsied as per protocol [9] (Figure 4). Anatomically, all four lobes of the liver namely, the right, left, caudate, and quadrate lobes was differentiated as mentioned by Harbin et al. [10]. Dimensions of fetal liver were measured, i.e., sagittal diameter (SD), transverse diameter (TD), and vertical length (VL) along with fetal liver volume (V) and fetal liver weight (W). The length and width of all four lobes, i.e., right lobe (RL, RW) (Figure 5), left lobe (LL, LW) (Figure 6), quadrate lobe (QL, QW) (Figure 7), and caudate lobe (CL, CW) (Figure 8) were measured. SD (cm) was the thickness of the liver between the two parallel vertical tangents passing through the anterior and posterior poles of the liver. VL (cm) was the maximum height of the liver between the horizontal plane passing through the upper and lowermost points of the liver (Figure 9). TD (cm) was the width of the liver, i.e., the maximum horizontal distance between the right and left borders of the liver, vertical planes passing through the right and left margins of the liver (Figure 8). RW was transverse distance measured from the most medial point of the right lobe to the falciform ligament. LW was the maximum transverse distance between the most medial points of the left lobe to the falciform ligament (porta hepatitis joins umbilical vein) (Figure 5). CL was the vertical distance between the superior margins of the liver to the porta hepatis. CW was measured from falciform ligament to lateral margin of inferior vena cava. QL was measured as the maximum vertical distance between the highest points on the tangents passing through superior and inferior borders of the quadrate lobe. QW was measured from the falciform ligament medially to the fossa of the gall bladder laterally. V was measured by the water displacement method and W by the BSA 224S-CW Analytical Balance (Sartorius AG, Göttingen, Germany). 

## Results

Data were arranged in a Microsoft Excel spreadsheet (Microsoft Corporation, Redmond, Washington, United States) to ensure that there is no data entry error. The values of each group were expressed as mean ± standard deviation. The samples were classified based on their gestation age, number, and gender (Table 1). Statistical analysis was done by calculating the mean, standard deviation, and p-value of ANOVA (Table 2, Table 3) of all the parameters followed by Pearson’s correlation (Table 4, Table 5, Table 6), and the Post Hoc test (Table 7, Table 8). Mean (in cm) and standard deviation of the fetal general morphometric parameters and its liver parameters were calculated according to gender in different gestational age groups (Table 9, Table 10). 

**Table 1:** Classification of fetal samples based on their groups, gestational age, and gender.

Group	Gestational age (weeks)	Male (N=27)	Female (N=6)	Total (N=33)
A	12-16	4	0	4
B	17-21	7	4	11
C	22-26	4	2	6
D	27-31	6	0	6
E	32-36	6	0	6

**Table 2:** Mean (in cm), standard deviation, and range of the fetal general morphometric parameters according to gestational age groups with P-value of ANOVA. FBW: Fetal body weight; HC: Head circumference; CC: Chest circumference; AC: Abdominal circumference; BPD: Bi-parietal diameter; ICD: Inner intercanthal distance; OCD: Outer intercanthal distance; HL: Hand length; FL: Foot length; CRL: Crown-rump length; CHL: Crown-heel length

Parameters (cm)	A (12-16 weeks)	B (17-21 weeks)	C (22-26 weeks)	D (27-31 weeks)	E (32- 36 weeks)	P-value (ANOVA)	Range (Group A-E)
FBW (g)	150±29.8	322.8±99	438±35.4	1000±605.4	2231±914	<0.001	110 - 3650
CRL (cm)	13.4 ± 2.8	19.5± 1.8	20.9 ± 2.3	29.3± 5.7	34.7± 4.5		10.5 - 40.4
CHL (cm)	19.2± 4.1	31.6± 3.6	31.1± 5.6	50.2± 13.4	57.3± 10.9		14.5 - 65.8
HL (cm)	1.3±0.6	2.8±0.5	2.9±0.4	4.2±0.6	5.14±0.6		0.8 -5.7
FL (cm)	1.6±0.54	3.4±0.8	3.3±0.9	5.±1.2	7.2±1.5		1 - 8.4
HC (cm)	11.1±2.1	17.8±1.9	18.2±2.6	26.3±6	31.1±4.3		9.5 - 34.5
CC (cm)	9.9±1.9	14.8±1.5	15.3±1.9	23.2±7.4	28± 5		8 -34.1
AC (cm)	8.7±1.2	13±1.42	13.7±1.2	21.9±6.9	26.7±3.9		7.5 -31.9
BPD (cm)	6.5±3.7	6.4±2.0	7.5±1	8.3±2.3	10.7±0.1	<0.002	4 - 12
ICD (cm)	0.6±.1	1.4±0.3	1.4±0.3	1.9±0.7	2.4±0.8	<0.001	0.5 -3.4
OCD (cm)	2.5±0.8	3.9±0.6	4±0.7	5.1±1.9	7.1±1		1.8 - 8.1

**Table 3:** Mean (in cm), standard deviation, and range of the fetal liver parameters according to gestational age groups with P-value of ANOVA. SD: Sagittal diameter; TD: Transverse diameter; VL: Vertical length; V: Fetal liver volume; W: Fetal liver weight; CL: Length of caudate lobe; CW: Width of caudate lobe; QL: Length of quadrate lobe; QW: Width of quadrate lobe; RL: Length of right lobe; RW: Width of right lobe; LL: Length of left lobe; LW: Width of left lobe

Parameters (cm)	A (12-16 weeks)	B (17-21 weeks)	C (22-26 weeks)	D (27-31 weeks)	E (32-36 weeks)	P-value (ANOVA)	Range (Group A-E)
W (g)	4.87±1.41	16.29±5.52	17.40±9.00	29.01±22.22	70.52±29.50	<0.001	2.9-103.4
V (ml)	5.0±1.22	12.07±5.64	12.90±7.87	23.10±19.53	64.40±30.33		3.5- 105
VL (cm)	1.10±0.21	1.60±0.33	1.55±0.43	1.65±0.26	2.24±0.28		0.8 - 2.5
TD (cm)	2.40±0.55	3.95±0.42	4.06±0.18	5.12±1.08	5.80±1.97		1.9 - 8.4
SD (cm)	1.85±0.37	3.17±0.43	3.28±0.77	3.95±1.14	5.82±1.50		1.6 - 7.4
CL (cm)	1.77±0.34	3.29±0.39	3.25±0.78	4.14±0.78	5.50±1.14		1.5 -7.0
CW (cm)	1.40±0.48	1.89±0.42	1.68±0.31	2.54±0.54	3.10±0.35		0.8 - 3.4
QL (cm)	1.52±0.17	3.05±0.40	3.30±0.80	3.84±0.71	5.46±1.58		1.3 - 7.2
QW (cm)	1.22±0.22	1.79±0.39	1.86±0.36	2.25±0.53	3.30±0.87		1 -4.3
RL (cm)	0.60±0.14	1.31±0.40	1.23±0.26	1.68±0.32	1.90±0.65		0.5 - 2.8
RW (cm)	0.37±0.09	0.90±0.28	0.85±0.42	1.25±0.37	1.46±0.40		0.3 - 1.8
LL (cm)	0.82±0.09	1.50±0.24	1.63±0.46	1.82±0.77	3.06±0.90		0.7 - 4.2
LW (cm)	0.40±0.11	0.92±0.31	0.95±0.23	1.02±0.30	1.4±0.50		0.3 - 2.1

**Table 4:** Pearson’s correlation in between fetal general parameters and fetal liver parameters p value < 0.001 except BPD. SD: Sagittal diameter; TD: Transverse diameter; VL: Vertical length; V: Fetal liver volume; W: Fetal liver weight; CL: Length of caudate lobe; CW: Width of caudate lobe; QL: Length of quadrate lobe; QW: Width of quadrate lobe; RL: Length of right lobe; RW: Width of right lobe; LL: Length of left lobe; LW: Width of left lobe; BPD: Bi-parietal diameter; GA: Gestational week; ICD: Inner inter-canthal distance; HC: Head circumference; CHL: Crown-heel length; CRL: Crown-rump length; FL: Foot length; HL: Hand length; CC: Chest circumference; AC: Abdominal circumference; BPD: Bi-parietal diameter

Parameters	GA (weeks)	CHL (cm)	CRL (cm)	FL (cm)	HL (cm)	HC (cm)	CC (cm)	AC (cm)	BPD (cm)	P-value (BPD)	ICD (cm)	OCD (cm)
W (g)	0.74	0.80	0.81	0.83	0.81	0.82	0.81	0.78	0.58	0.01	0.81	0.83
V (ml)	0.71	0.77	0.79	0.81	0.78	0.78	0.79	0.75	0.58		0.79	0.82
VL (cm)	0.79	0.80	0.82	0.83	0.84	0.82	0.80	0.80	0.51	0.02	0.80	0.81
TD (cm)	0.77	0.70	0.77	0.72	0.76	0.73	0.72	0.68	0.51	0.01	0.77	0.74
SD(cm)	0.61	0.68	0.69	0.74	0.66	0.70	0.69	0.63	0.43	0.02	0.70	0.71
CL (cm)	0.70	0.70	0.67	0.70	0.73	0.70	0.66	0.61	0.28	0.13	0.69	0.63
CW (cm)	0.67	0.66	0.65	0.69	0.68	0.67	0.64	0.60	0.35	0.05	0.70	0.61
QL (cm)	0.73	0.72	0.74	0.76	0.77	0.76	0.74	0.70	0.52	0.01	0.71	0.74
QW (cm)	0.62	0.62	0.59	0.65	0.67	0.63	0.58	0.56	0.30	0.10	0.61	0.61
RL (cm)	0.79	0.83	0.83	0.87	0.86	0.86	0.82	0.80	0.50	0.03	0.83	0.83
RW (cm)	0.79	0.84	0.83	0.84	0.82	0.82	0.79	0.83	0.54	0.01	0.79	0.82
LL (cm)	0.79	0.82	0.83	0.83	0.85	0.85	0.82	0.78	0.50		0.78	0.80
LW (cm)	0.77	0.80	0.81	0.82	0.84	0.81	0.80	0.79	0.46	0.05	0.73	0.78

**Table 5:** Pearson’s correlation between fetal general parameters with p-value <0.001 except BPD. FBW: Fetal body weight; HC: Head circumference; CC: Chest circumference; AC: Abdominal circumference; BPD: Bi-parietal diameter; ICD: Inner intercanthal distance; OCD: Outer intercanthal distance; HL: Hand length; FL: Foot length; CRL: Crown-rump length; CHL: Crown-heel length; GA: Gestational age; BPD: Bi-parietal diameter

Parameters	GA (weeks)	CHL (cm)	CRL (cm)	FL (cm)	HL (cm)	HC (cm)	CC (cm)	AC (cm)	BPD (cm)	P-value (BPD)	ICD (cm)	OCD (cm)
GA(weeks)	1	.87^*^	0.91	0.86	0.92	0.88	0.85	0.87	0.59	0.01	0.74	0.79
CHL (cm)	0.87	1	0.98	0.98	0.93	0.98	0.96	0.96	0.52	0.02	0.82	0.84
CRL (cm)	0.92	0.98	1	0.97	0.95	0.98	0.98	0.97	0.56	0.01	0.85	0.85
FL (cm)	0.86	0.98	0.97	1	0.93	0.99	0.98	0.96	0.52	0.01	0.84	0.85
HL (cm)	0.92	0.93	0.95	0.93	1	0.94	0.90	0.90	0.59	0.01	0.86	0.87
HC (cm)	0.88	0.98	0.98	0.99	0.94	1	0.99	0.97	0.53	0.02	0.83	0.84
CC (cm)	0.85	0.96	0.97	0.98	0.89	0.99	1	0.98	0.49	0.04	0.80	0.78
AC (cm)	0.87	0.96	0.97	0.96	0.90	0.97	0.98	1	0.50	0.03	0.79	0.80
BPD (cm)	0.59	0.52	0.52	0.52	0.59	0.53	0.49	0.50	1	-	0.63	0.71
ICD (cm)	0.74	0.82	0.85	0.84	0.85	0.83	0.80	0.79	0.63	0.01	1	0.94
OCD (cm)	0.79	0.84	0.85	0.85	0.87	0.84	0.80	0.80	0.71	0.01	00.94	1

**Table 6:** Pearson’s correlation between fetal liver parameters. SD: Sagittal diameter; TD: Transverse diameter; VL: Vertical length; V: Fetal liver volume; W: Fetal liver weight; CL: Length of caudate lobe; CW: Width of caudate lobe; QL: Length of quadrate lobe; QW: Width of quadrate lobe; RL: Length of right lobe; RW: Width of right lobe; LL: Length of left lobe; LW: Width of left lobe (LW)

Parameters (cm)	W (g)	LV (ml)	VL	TD	SD	CL	CW	QL	QW	RL	RW	LL	LW
W (g)	1	0.99	0.96	0.66	0.74	0.72	0.71	0.93	0.76	0.94	0.75	0.92	0.89
LV (ml)	0.99	1	0.94	0.66	0.73	0.69	0.69	0.89	0.72	0.89	0.73	0.89	0.86
VL (cm)	0.96	0.94	1	0.67	0.76	0.80	0.76	0.93	0.80	0.97	0.78	0.96	0.90
TD (cm)	0.66	0.66	0.67	1	0.56	0.66	0.55	0.62	0.45	0.69	0.69	0.73	0.59
SD (cm)	0.74	0.73	0.76	0.56	1	0.59	0.64	0.68	0.64	0.76	0.70	0.74	0.74
CL (cm)	0.72	0.70	0.80	0.67	0.60	1	0.83	0.79	0.75	0.82	0.66	0.83	0.73
CW (cm)	0.72	0.69	0.76	0.56	0.64	0.83	1	0.80	0.78	0.82	0.56	0.75	0.61
QL (cm)	0.93	0.90	0.93	0.62	0.68	0.79	0.80	1	0.85	0.92	0.61	0.92	0.85
QW (cm)	0.76	0.72	0.80	0.45	0.64	0.75	0.78	0.85	1	0.81	0.50	0.81	0.73
RL (cm)	0.94	0.90	0.97	0.70	0.76	0.82	0.82	0.92	0.81	1	0.78	0.93	0.87
RW (cm)	0.75	0.73	0.78	0.69	0.70	0.66	0.56	0.61	0.50	0.77	1	0.76	0.76
LL (cm)	0.92	0.89	0.96	0.73	0.74	0.83	0.75	0.92	0.81	0.93	0.76	1	0.87
LW (cm)	0.89	0.86	0.90	0.59	0.75	0.73	0.61	0.85	0.73	0.87	0.76	0.87	1

**Table 7:** Post-hoc test in general fetal morphometric parameters within the groups. FBW: Fetal body weight; HC: Head circumference; CC: Chest circumference; AC: Abdominal circumference; BPD: Bi-parietal diameter; ICD: Inner intercanthal distance; OCD: Outer intercanthal distance; HL: Hand length: FL: Foot length; CRL: Crown-rump length; CHL: Crown-heel length; GA: Gestational age

Parameters	Mean difference within the groups									
	A-B	A-C	A-D	A-E	B-C	B-D	B-E	C-D	C-E	D-E
CHL (cm)	-12.4	-12.0	-31.0	-38.2	.45	-18.7	-25.8	-19.1	-26.2	-7.2
CRL (cm)	-6.05	-7.6	-16	-21.3	-1.5	-9.9	-15.3	-8.4	13.8	-5.39
FL (cm)	-1.8	-1.7	-4.08	-5.6	.15	-2.4	-3.8	-2.5	-3.9	-1.4
HL (cm)	-1.5	-1.6	-3	-3.9	-0.03	-1.41	-2.34	-1.4	-2.4	-1
HC (cm)	-6.7	-7.1	-15.2	-20.2	-0.5	-8.5	-13.3	-8.1	-12.9	-4.8
CC (cm)	-4.9	-5.4	-13.3	-18.1	-0.5	-8.4	-13.2	-7.9	-12.8	4.8
AC (cm)	-4.3	-4.9	-12.3	-17.9	-0.65	-8.05	-13.6	-7.4	-13	-5.5
BPD (cm)	0.11	-1.1	-1.9	-4.2	-1.2	-2.0	-4.4	-0.9	-3.2	-2.4
ICD (cm)	-0.8	-0.8	-1.32	-1.8	.03	-0.6	1.0	-0.6	-1.0	-0.5
OCD (cm)	-1.5	-1.6	-2.7	-4.6	-0.2	-1.3	-3.2	-1.06	-3	-2.0

**Table 8:** Post-hoc test in fetal liver parameters within the groups. SD: Sagittal diameter; TD: Transverse diameter; VL: Vertical length; V: Fetal liver volume; W: Fetal liver weight; CL: Length of caudate lobe; CW: Width of caudate lobe; QL: Length of quadrate lobe; QW: Width of quadrate lobe; RL: Length of right lobe; RW: Width of right lobe; LL: Length of left lobe; LW: Width of left lobe

Parameters	Mean difference within the groups									
	A-B	A-C	A-D	A-E	B-C	B-D	B-E	C-D	C-E	D-E
W (g)	-11.4	-12.6	-24.2	-65.7	-1.1	-12.8	-54.3	-11.6	-53.2	-41.5
LV (ml)	-7.1	-8	-18.2	-60.0	-0.9	-11.1	-52.4	-10.2	51.5	-41.3
VL (cm)	-1.32	-1.5	-1.6	-4.1	-0.2	-0.4	-2.8	-0.3	-2.6	-2.4
TD (cm)	-1.6	-1.7	-2.8	-3.4	-0.2	-1.2	-1.9	-1.1	-1.8	-0.7
SD (cm)	-0.5	-0.5	-0.6	-1.2	0.05	-0.05	-0.7	-0.2	-0.7	-0.6
CL (cm)	-0.7	-0.6	-1.1	-1.3	0.09	-0.4	-0.6	-0.5	-0.7	-0.3
CW (cm)	-0.6	-0.5	-0.9	-1.1	0.05	-0.4	-0.6	-0.4	-0.60	-0.2
QL (cm)	-0.7	-0.9	-1	-2.3	-0.4	-0.3	-1.6	-0.2	1.5	-1.3
QW (cm)	-0.6	0.6	-0.7	-1.04	-0.02	-0.10	-0.6	-0.1	-0.5	-0.5
RL (cm)	-1.6	-1.5	-2.4	-3.8	0.05	-0.10	-2.3	-0.9	-2.3	-1.4
RW (cm)	-0.5	-0.3	-1.2	-1.8	0.2	-0.7	-1.2	-0.9	-1.4	-0.6
LL (cm)	-1.6	-0.3	-2.3	-3.9	-0.3	-0.8	-2.4	-0.6	-2.2	-1.6
LW (cm)	-0.36	-0.7	-1.03	-2.1	-0.1	-0.5	-1.5	-0.4	-1.5	-1.1

**Table 9:** Mean (in cm) and standard deviation of the fetal general morphometric parameters according to gender in different gestational age groups. FBW: Fetal body weight; HC: Head circumference; CC: Chest circumference; AC: Abdominal circumference; BPD: Bi-parietal diameter; ICD: Inner intercanthal distance; OCD: Outer intercanthal distance; HL: Hand length; FL: Foot length; CRL: Crown-rump length; CHL: Crown-heel length; GA: Gestational age

Parameters	A (12-16 weeks)	B (17-21 weeks)		C (22-26 weeks)		D (27-31 weeks)	E (32-36 weeks)
	Male (N=4)	Male (N=7)	Female (N=4)	Male (N=4)	Female (N=2)	Male (N=6)	Male (N=6)
GA (weeks)	15.5 ± 0.6	19.0±1.8	19.5±1	25.0±1.5	23.3±0.5	30.6± 2.7	34.2±1.5
CHL (cm)	19.2 ±4.1	30.5±4.2	32.07±3.3	33.6±7.3	29.9±5.3	50.2± 13.4	57.3± 10.9
CRL (cm)	13.4 ±2.8	19.3±2.6	19.5±1.3	21.8±3.3	21.0±2.2	29.3± 5.7	34.7±4.6
FL (cm)	1.7 ±0.6	3.3±1.0	3.5±0.6	3.5±1.5	3.2±0.8	5.8± 2.0	7.2±1.5
HL (cm)	1.3± 06	3.0±0.6	2.7±0.5	3.2±0.3	2.7±0.2	4.3± 0.6	5.1±0.6
HC (cm)	11.1 ± 2.1	17.2±2.	18.1±1.3	18.4±3.4	18.1±2.6	26.3±6.7	31±4.3
CC (cm)	9.9± 1.9	14.5±2.3	15.0±1.0	15.7±3.1	15.1±1.5	23.2±7.4	28.0±5.0
AC (cm)	8.7± 1.2	43.4±61.1	13.0±1.5	13.2±1.6	13.9±1.1	21.9± 7.0	26.6±3.9
BPD (cm)	6.5± 3.6	6.9±2.5	6.1±1.8	7.6±0.7	7.5±1.2	8.3± 2.2	10.7±1.0
ICD (cm)	0.6± 0.1	1.6±0.3	1.3±0.3	1.4±0.5	1.4±0.3	1.9±0.6	2.4±0.8
OCD (cm)	2.5± 0.8	4.8±0.5	3.7±0.6	4.1±1.3	4.0±0.4	5.1± 1.9	7.1±1.0

**Table 10:** Mean (in cm) and standard deviation of the fetal liver parameters according to gender in different gestational age groups. SD: Sagittal diameter; TD: Transverse diameter; VL: Vertical length; V: Fetal liver volume; W: Fetal liver weight; CL: Length of caudate lobe; CW: Width of caudate lobe; QL: Length of quadrate lobe; QW: Width of quadrate lobe; RL: Length of right lobe: RW: Width of right lobe; LL: Length of left lobe; LW: Width of left lobe

Parameter	A (12-16 weeks)	B (17-21 weeks)		C (22-26 weeks)		D (27-31 weeks)	E (32-36 weeks)
	Male (N=4)	Male (N=7)	Female (N=4)	Male (N=4)	Female (N=2)	Male (N=6)	Male (N=6)
W (g)	4.9± 1.5	15.1±5.5	17.1±5.9	19.4±7.7	16.5±10.6	29.1± 22.2	70.5±29.5
V (ml)	5.0± 1.3	10.1±4.3	13.3±6.3	16.0±5.6	11.4±9.1	23± 19.6	64.4±30.3
VL (cm)	1.9± 0.4	3.0±3.0	7.4±10.9	3.6±0.8	3.2±0.9	4.0± 1.2	5.8±1.6
TD (cm)	2.5± 0.6	4.1±0.5	3.9±0.5	4.2±0.4	4.1±0.1	5.2± 1.1	5.8±2.0
SD (cm)	1.1± 0.3	1.5±0.3	1.7±0.4	1.5±0.3	1.6±0.6	1.7± 0.3	2.3±0.3
CL (cm)	0.6± 0.2	1.6±0.4	1.2±0.5	1.2±0.5	1.3±0.3	1.7±0.4	2.0±0.7
CW (cm)	0.4± 0.1	1.0±0.3	0.9±0.3	1±0.8	0.9±0.4	1.3±0.4	1.5±0.5
QL (cm)	0.9± 0.1	1.5±0.3	1.5±0.2	1.8±0.8	1.6±0.4	1.9±0.8	3.1±1.0
QW (cm)	0.4± 0.2	1.0±0.4	0.9±0.3	0.9±0.3	1.1±0.3	1.1±0.4	1.5±0.6
RL (cm)	1.8± 0.4	3.2±0.5	3.3±0.4	3.4±1.0	3.2±0.9	4.2±1.3	5.5±1.2
RW (cm)	1.4± 0.5	2.0±0.5	1.9±0.4	1.8±0.3	1.7±0.4	2.5±0.6	3.1±0.4
LL (cm)	1.6±0.2	3.0±0.6	3.2±0.4	3.4±0.8	3.3±1.0	3.9±0.8	5.5±1.6
LW (cm)	1.3±0.3	1.9±0.6	1.88±0.4	1.9±0.3	1.9±0.5	2.3±0.6	3.3±0.9

The mean ± standard deviation of gestational age (weeks) of both males and females was 24.30 ± 6.7. Liver weight was 2.9 g in 15 weeks, and it increased consistently to 90g in 36 weeks. The correlation coefficient of FBW to gestational age was R= 0.74, p-value <0.001. Pearson’s correlation was applied amongst all the fetal general morphometric and liver morphometric parameters. The correlation was found to be statistically significantly correlated with all the parameters with a p-value <0.001 except for BPD. Therefore, change in general fetal morphometrics except BPD is directly proportional to changes in the morphometric parameters of the liver. 

All the fetal general and liver morphometric parameters were correlated by Pearson’s correlation to document variability between continuous variables namely, gestational age, CRL, CHL, HL, FL, HC, AC, BPD, CC, ICD, and OCD. It was seen that there was a strong correlation between fetal gestational age with all the morphometric parameters with p-value < 0.001 except BPD, R=0.59, p-value >0.001.

So, we can conclude that all the fetal general parameters and fetal liver dimensions were statistically significantly correlated with each other except BPD, which was a weak correlation factor to estimate the fetal gestational age with variable p-value ranging from 0.00 to 0.81. The correlation coefficient was > 60% for gestational age, CRL, CHL, HL, FL, AC, ICD, OCD, HC, and CC except for BPD with a correlation coefficient of < 60%. Therefore, any change in values of all morphometric parameters of the fetus and its liver parameters was directly proportional to each other and vice versa except with BPD with a p-value <0.001.

## Discussion

Agnihotri et al. estimated gestation age using fetal liver morphometry of gestational age 13-32 weeks [11]. Age distribution was done on basis of CRL (mm), i.e., month four (CRL 61-100 mm), month five (CRL 101-150 mm), month six (CRL 151-200 mm), month seven (CRL 201-260 mm), month eight (CRL 261-320 mm). The regression coefficients were obtained between CRL and RL, LL, RW, LW, SD, and W. A strong correlation was observed between liver morphometry and CRL. These findings coincide with the present study. Any variation in results of the normal fetal liver can be used to determine liver pathologies due to intrauterine growth restrictions, etc. 

Hosapatna et al. performed a study on morphometry of caudate and quadrate lobe of liver on 18 samples of gestational age 12-36 weeks [7]. The mean ± standard deviation (mm) of the length of the quadrate lobe was 21.5±5.5 mm, width of the quadrate lobe 11.1 ± 3.5mm, length of the caudate lobe 15.6±5.3 mm, and width of the caudate lobe was 10.2±2.6 mm. Pearson’s correlation was applied between gestational age and length and width of the quadrate lobe (0.8 and 0.4 respectively, p-value: <0.001 and 0.03). These findings were in accord with the current study.

Archie et al. reviewed the Quantitative Standards for Fetal and Neonatal Autopsy and concluded a reference chart for 12-44 weeks of gestational age of parameters (in mm): CHL, CRL, AC, HC, CC, BPD, FBW, ICD, OCD, HL, FL [12]. They also observed inter nipple distance (IND), philtrum length (PL), small intestine length (SIL), and large intestine length (LIL). The parameters (range) from 12-36 weeks were CHL: 93.0 ± 9.7 to 444 ± 44, CRL: 76.1 ± 6.7 to 318 ± 33, BPD: 19.5 ± 3.5 to 87.8 ± 5.2, HC: 67.7 ± 11.6 to 319 ± 16, OCD: 14.0 ± 4.0 to 61.0 ± 4.3, ICD: 6.04 ± 1.55 to 20.3 ± 1.8, CC: 76.7 to 292, FL: 9.74 ± 1.11 to 69.2 ± 6.7. In the current study, our calculations differences were due to anatomical varieties and racial reasons. 

Szpinda et al. assessed three-dimensional growth dynamics of the human fetal liver of gestational age 18-30 weeks [13]. He assessed the VL, SD, and TD of the liver. The mean transverse diameter at 18 weeks was 19.5±1.02mm and 39.65±7.05mm at 30 weeks. The mean transverse diameter at 18 weeks was 29.44±3.73mm to 53.13±5.31mm at 30 weeks. The mean sagittal diameter at 18 weeks was 22.97±3.79 to 43.22±5.49 mm at 30 weeks. The Pearson's correlation between gestational age and crown-rump length was 0.99 and the p-value <0.001. Similar findings were elicited in our study. This can be used to distinguish the abnormal from normal development of the fetal liver.

Tongprasert et al. conducted a study to create a normal reference chart between fetal liver length (through ultrasound) and gestational age ranging from 14-40 weeks [14]. The reference chart is of clinical use in cases of fetal anemia. They observed a substantial increase in fetal liver length with an increase in gestational age. The results obtained in the current study are in accord with their findings.

Albay et al. assessed the morphological development of the human fetal liver of gestational age 9-40 weeks [15]. The CRL ranged from 70-420 mm. The obtained samples were grouped based on shapes, i.e., trapezoid, triangular, square, and rectangle. In terms of gestational ages, the obtained fetuses were categorized as group A (9-12 weeks), group B (13-25 weeks), group C (26-37 weeks), and group D (38-40 weeks). The mean liver weight increased from 1.8 ± 0.7 in group A to 74 ± 20.3 in group D. The mean liver volume increased from 1.8 ± 0.6 in 157 group A to 71.8 ± 19.9 in group D. The mean liver width ranged between 19 ± 3 mm in group A to 82 ± 9 mm in group D. Mean liver thickness ranged between 11 ± 1 mm in group A to 31 ± 8 mm in group D. The mean liver height ranged between 13 ± 2 in group A to 47 ± 8mm in group D. The Pearson’s correlation coefficient between liver volume and gestation age was R = 0.86 and p-value < 0.05), while the widths of the caudate and quadrate lobes, as well as the height of the quadrate lobe, demonstrated a significant increase (p-value <0.05). This can be used as a valuable tool to assess intrauterine infections, isoimmunization, fetal heart failure, neoplasms, and fetal macrosomia, metabolic disorders like fetal anemia [11].

Chang assessed the normal fetal liver volume by three-dimensional ultrasound with gestational age to establish a normal reference chart for clinical use [16]. The results obtained were statistically significantly correlated with fetal biometry namely, estimated FBW, BPD, femur length, HC, occipitofrontal diameter, and abdominal circumference (for all, p-value <0.001). In our study, we also found a strong statistically significant correlation between V and other morphometric parameters with gestational age. 

Roberts et al., in research, measured the length of the liver in small-for-gestational-age fetuses through ultrasonography [17]. Their study aims to reflect the correlation between small abdominal circumferences with the fetal liver. In intrauterine growth restriction (IUGR), the right lobe of the liver is thought to be more severely impacted than the left lobe; hence, they measured right lobe length. They concluded that the small circumference of the abdomen reflects the reduced growth of intra-abdominal organs, less fat, and poor growth of lungs resulting in the elevated diaphragm.

In the current study, the range of gestational age was 12-36 weeks. Pearson’s correlation was calculated for all the general morphometric parameters of the fetus. All the correlations were statistically significant, p-value <0.001 except for BPD. The correlation coefficient between VL (cm) and BPD was r = 0.51, p-value <0.02, gestational age and BPD (R= 0.59), p-value = >0.001, BPD and HC is R=0.53, p-value <0.02, BPD and AC is R = 0.50, p-value =<0.03. All the other correlations were statistically significant, p-value <0.001. The results show a statistically significant rise in V from group A to group E. These findings help to evaluate IUGR and other pathological conditions affecting fetal growth.

Limitations of the study

The morphometric analysis of the fetal liver with respect to its gestational age was done only on basis of gross morphological features, as the shape of the fetal liver was not included. In further studies, the research can be broadened by observing the morphological appearance of cells, distribution per unit area, and volumetry in a large sample size. The authors recommend further studies with a large sample size including specimens from specific diseases and those exposed to teratogenic factors. 

## Conclusions

A strong statistically significant correlation was found between CHL, AC, CRL, HL, FL, HC, CC, V, W, and gestational age, p-value <0.001. Statistically, a significant increase was found amongst all the fetal general parameters and parameters of the liver except BPD. BPD was statistically not significant with CC, CHL, CL, CW, QW, LW with p-value 0.02, 0.02 and 0.13, 0.05, 0.10, and 0.05. It is weakly correlated with all other parameters. The knowledge of anatomical details of fetal helps in the diagnosis of liver anomalies, fetal macrosomia, gestational diabetes, Down's syndrome, etc. It plays a key role to achieve a correct preoperative diagnosis and will help the surgeon in planning biliary surgeries or portosystemic anastomoses. 
